# Editorial: Plant responses to salt stress

**DOI:** 10.3389/fpls.2024.1475599

**Published:** 2024-09-02

**Authors:** Zulfiqar Ali Sahito, Adalberto Benavides-Mendoza, Keni Cota-Ruiz

**Affiliations:** ^1^ Industrial Crops Research Institute, Yunnan Academy of Agricultural Sciences (YAAS), Kunming, Yunnan, China; ^2^ Universidad Autónoma Agraria Antonio Narro, Department of Horticulture, Saltillo, Mexico; ^3^ Biology Department, Utica University, Utica, NY, United States

**Keywords:** agriculture, salt stress, salt tolerance, antioxidants, osmolyte accumulation

As the world population is increasing, food production largely depends on agriculture. However, crops are under pressure from biotic and abiotic stressors such as climate change, excess agrochemicals, pests, diseases, and soil salinization. The expansion of irrigation practices has led to higher salt contents in agricultural lands, impairing plant function and reducing yields. Hence, investigating how plants behave against salt stress is imperative. Furthermore, a clearer mechanistic understanding of those responses is fundamental to leveraging proactive responses and developing technologies to improve plant fitness and productivity. The articles published in our Research Topic summarize cutting-edge research on the effects of salt stress on several plant species. These studies explore the genetic, physiological, and biochemical responses of plants to salt stress. Below, we contextualize the findings of each article and briefly discuss their impact.

Taybi and Alyahya examined how bread wheat plants cope with salt in their roots and shoots. A comparative RNA sequencing analysis was conducted on bread wheat (Najran cultivar) under unstressed and salt-stressed conditions. The authors reported that more genes were affected in the roots than in the shoots. This study highlighted the potential involvement of glutathione metabolism, secondary metabolite biosynthesis, and galactose metabolism in wheat salt tolerance. These findings could help scientists find ways to make wheat more tolerant to salt, which would be very important for growing enough food.

Meena et al. investigated the potential of the ACC deaminase producer Nocardioides sp. in enhancing wheat growth in saline environments. This research aimed to elucidate the molecular mechanisms that control Nocardioides sp.-facilitated salinity tolerance in wheat. The authors reported that Nocardioides sp. inoculation improved wheat growth under saline conditions and increased biomass. It also increased antioxidant potential and promoted elevated stress-responsive gene transcripts. These findings suggest the potential of Nocardioides sp. to increase wheat tolerance to salinity stress, with implications for agricultural sustainability in saline-prone regions.

Zhang et al. provided a thorough overview of how *Dunaliella salina* responds to salt stress at the transcriptomic level, highlighting the major cellular biological processes that are regulated. The authors revealed the extensive upregulation of genes involved in DNA repair, protein folding, and cell redox homeostasis in response to salt stress. As noted by the authors, these findings indicate that *D. salina* has developed important mechanisms to survive under salt stress, which were previously overlooked by other researchers. This study encourages a need for further in-depth study in this area.

Ma et al. sequenced libraries prepared from *Suaeda dendroides* plants subjected to both optimal salt and high salt concentrations. This approach resulted in the discovery of differentially expressed genes. The data revealed significant upregulation of genes associated with plant hormone signal transduction, cell wall biosynthesis and modification, organic osmolyte accumulation, ion homeostasis, and reactive oxygen species detoxification in response to high-salt treatment. The relevance of this study lies in its contribution to our understanding of how halophytes adapt to high salinity.

Xu et al. researched the effectiveness of the phytochemical eugenol in promoting salt tolerance in tobacco seedlings through histochemical, physiological, and biochemical methods. They found that eugenol reduces the obstructing effects of salt stress on seedling growth in a dose-dependent manner and that this effect is optimal at 20 µM. Eugenol treatment reduced the accumulation of reactive oxygen species, lipid peroxidation, and osmoprotectant content in salt-stressed seedlings. This study showed that eugenol can increase plant growth in salty soils. This could lead to new ways to help plants thrive in harsh conditions.

Rajabi Dehnavi et al. explored the responses of ten sorghum genotypes to varying salt stress levels. They successfully identified key indicators for salt tolerance, including the K/Na ratio, MDA, MSI, and proline contents, and SOD activity, which proved to be effective in differentiating between tolerant and sensitive genotypes. These findings provide valuable insights for sorghum breeding. The authors emphasized the potential for enhancing salt tolerance in sorghum to support sustainable production in saline environments. The identified salt-tolerant sorghum genotypes, such as Pegah and GS4, are promising candidates for further evaluation in salt-affected environments, with potential benefits for agriculture and food security.

Song et al. identified salt-tolerant and salt-sensitive genotypes of smooth bromegrass. The salt-tolerant genotype, Q25, presented a better performance in diverse characteristics, such as leaf relative water content, photosynthetic performance, and proline content, compared to the salt-sensitive genotype, Q46. KEGG analysis identified genes involved in plant hormone signal transduction and the MAPK signaling pathway responsible for the salt response differences between the two genotypes. This study also uncovered candidate genes associated with salt tolerance, including zinc finger transcription factors. This research offers valuable insights into critical genes linked to salt tolerance, which could help develop salt-tolerant crop varieties to address the adverse effects of salinity on crop yield.

Mi et al. demonstrated distinctive responses of *Astragalus membranaceus* and *Medicago sativa* during seedling growth under salt stress. Their study revealed that salinity directly affects plant characteristics and physiological indices and indirectly impacts leaf succulence. Additionally, the study noted variations in the activity of specific protective enzymes in response to salt stress between the two legumes. This research has implications for agriculture because it helps us understand how legume species respond to salt stress. This is particularly interesting in the context of restoring and utilizing salinized grasslands.

Li et al. examined the genetic factors affecting salt tolerance in maize. They found a specific allele, ZmSC IL76, derived from *Zea perennis*, which increased salt tolerance in maize. This allele had a non-synonymous mutation that improved salt tolerance compared to the ZmSC Z58 allele. Additionally, this study identified ZmSC as a potential regulator of salt tolerance pathways, notably playing a role in controlling ABA content and downstream gene expression. These findings offer valuable insights for further molecular breeding and genetic engineering research to enhance salt tolerance in maize and likely other crops.

Tang et al. focused on assessing the ability of *Miscanthus sacchariflorus* and *M. lutarioriparius* to grow in saline soils for bioenergy production. This study revealed a wide range of salt tolerances among different genotypes, with salt-tolerant variants exhibiting relatively low Na+ levels and a positive relationship between K^+^ and Na^+^ contents. Additionally, the research highlighted the different mechanisms these plants employ to adapt to salt stress, including regulating ion balance, increasing K^+^ absorption, excluding Na^+^ from the shoot, and storing Na+ in shoot vacuoles. By establishing a mini-core elite collection for salt tolerance, this study provides a valuable gene pool for future investigations of salt tolerance mechanisms in Miscanthus.

Duan et al. revealed that tetraploid *Plumbago auriculata* plants have an increased tolerance to salt by selectively secreting more sodium (Na^+^) compared to diploids when exposed to salt stress. This increased Na+ secretion in tetraploids was associated with increased salt gland calcium (Ca^2+^) content. The research also investigated the effects of the addition of calcium, inhibition of H_2_O_2_ generation, and H^+^-ATPase activity on sodium and potassium (K^+^) secretion rates in both diploid and tetraploid plants under salt stress. These findings provide insights into the mechanisms of salt tolerance in plants, particularly how tetraploid plants may adapt to salt stress by enhancing selective sodium secretion through the modulation of salt gland calcium content.

